# Profile of maladaptive and normative eating behaviors in correlation with rumination: a cross-sectional study among Lebanese adults

**DOI:** 10.1186/s41155-024-00334-x

**Published:** 2025-01-03

**Authors:** Emmanuelle Awad, Diana Malaeb, Mirna Fawaz, Lara Youssef, Anna Brytek-Matera, Souheil Hallit, Sahar Obeid

**Affiliations:** 1 Department of Psychology and Education, School of Arts and Sciences, Lebanese American University, Beirut, Lebanon; 2 College of Pharmacy, Gulf Medical University, Ajman, United Arab Emirates; 3 College of Health Sciences, American University of the Middle East, Kuwait, Kuwait; 4 Department of Medical Laboratory Sciences, Faculty of Health Sciences, University of Balamand (UOB), Balamand, Lebanon; 5 Institute of Psychology, University of Wroclaw, Dawida 1, Wroclaw, 50-527 Poland; 6 School of Medicine and Medical Sciences, Holy Spirit University of Kaslik, P.O. Box 446, Jounieh, Lebanon; 7 Applied Science Research Center, Applied Science Private University, Amman, Jordan; 8 Department of Psychology and Education, School of Arts and Sciences, Lebanese American University, Jbeil, Lebanon; 9 Psychology Department, Effat University, College of Humanities, Jeddah, 21478 Saudi Arabia

**Keywords:** Maladaptive eating, Normative eating, Rumination, Repetitive negative thinking, Lebanon

## Abstract

**Background:**

Dieting is a common practice around the world. People who wish to lose weight, improve their eating habits, or reach a desired level of health often diet. Rumination, a pattern of repetitive negative thoughts and emotions, is typically found when individuals diet. The current study aimed to identify physical and psychological differences between maladaptive and normative eating behaviors in a sample from Lebanon.

**Methods:**

A cross-sectional design was adopted for the current study. It occurred between June and July 2022. Four hundred participants aged 18 and above participated. The research team used a snowball sampling method to recruit volunteers from all governorates of Lebanon.

**Results:**

Beta values were used to compare independent variables in order to infer those that have the strongest effect on the dependent ones. Higher Body Mass Index, and belonging to cluster 3 (restriction of food intake and rumination) and cluster 1 (maladaptive eating behavior and rumination) compared to cluster 2 (normative eating behavior and thought) were significantly related to more dieting. Higher physical activity index and belonging to cluster 3 (restriction of food intake and rumination) and cluster 1 (maladaptive eating behavior and rumination) compared to cluster 2 (normative eating behavior and thought) were significantly related to higher levels of orthorexia nervosa tendencies.

**Conclusions:**

The current research demonstrated a relationship between Body Mass Index, physical activity, rumination, and maladaptive eating patterns including restriction of food intake, dieting, and orthorexia nervosa. These results can help with identifying physical and psychological factors associated with maladaptive eating patterns, as well as guide interventions within the Lebanese population.

## Background

Eating patterns and dieting styles are a prominent concern for the public health domain. Certain countries employ governmental agencies to develop recommendations and guidelines for a healthy eating lifestyle (Yang et al., 2018). According to previous research, eating styles in the Mediterranean are described as generally healthy and even used as a reference for healthy eating (Bamia et al., 2017; Boucher, 2017; Monteagudo et al., 2015). Having said that, further investigations are required to determine optimal eating styles, as well as its association with psychological variables (Adan et al., 2019).

### Eating behaviors

Dieting is typically done to achieve a desired weight or body; it could be defined as the restriction of food quantity or certain foods in order to achieve a desirable weight or body type (Markey & Meghan M. Gillen., 2023). It has been previously established through research that methods of dieting were associated with maladaptive eating patterns (Levinson et al., 2020). Following a special diet including specific restrictions was not only related to eating disorders, but also orthorexia nervosa (Plichta & Jezewska-Zychowicz, 2020), an eating pattern that consists of adhering to strict nutritional rules and consumption of foods that are perceived as healthy (Donini et al., 2022; Niedzielski & Kaźmierczak-Wojtaś, 2021). According to the literature, some studies hypothesized that orthorexia nervosa is an eating disorder or a symptom of a psychiatric disorder (Costa et al., 2017), while other studies questioned that it might be related to obsessive–compulsive disorder (A. J. A. o. P. Brytek-Matera & psychotherapy, 2012). Overall, it seems that a pattern of restraint in eating behaviors is the common pathway of eating patterns becoming maladaptive eating behaviors (Morrissey et al., 2020).

### Rumination and eating behaviors

Another considerable aspect of maladaptive eating patterns is rumination, recurring negative thoughts about emotions and experiences related to oneself (Watkins, 2008). It has been shown in the past that having repetitive negative thoughts contributed to dieting and lack of control in food consumption (Waliłko et al., 2021), worsening psychopathology (Watkins & Roberts, 2020), and higher maladaptive eating patterns characterized by restriction of food intake (Smith et al., 2018) (Nolen-Hoeksema & Watkins, 2011). Having said that, compared to research conducted on the relationship between eating behaviors and other psychological variables, the ones investigating the association with rumination are somewhat few (Smith et al., 2018).

### Sociodemographic factors and eating behaviors

As for the associations between sociodemographic factors and eating patterns, the literature offers important observations. People engaging in maladaptive eating patterns, including restriction of food intake, showed a higher probability of having a greater Body Mass Index (BMI) than those who do not (Yilmaz et al., 2019). The population of individuals who restrict food intake has a higher prevalence of women than men, where women focused more on dieting for weight loss and maintaining body thinness (Contois, 2019) (Brown et al., 2020). In general, dietary behaviors differ among genders in how and what they eat (Grzymisławska, Puch, Zawada, & Grzymisławski, 2020). Socioeconomic status (SES) also impacted the association between gender and maladaptive eating behaviors; men with lower SES and women with low and high SES engaged in more maladaptive eating behaviors (Lee et al., 2013), while other studies found that people with higher monetary income showed a lower probability of engaging in problematic eating behaviors, with no gender differences (Gedeon et al., 2022; Mei et al., 2022). On the other hand, the relationship between BMI and orthorexia is controversial; while one study showed no evidence connected BMI with the probability of developing ON (Varga et al., 2013), another one showed a positive association (M. Sfeir et al., 2022). Moreover, findings suggest that eating patterns, BMI, and physical activity are interrelated (Radwan et al., 2019). As for results related to Lebanon, orthorexia nervosa was correlated to worse psychological outcomes, including quality of life, as well as restriction of food intake (E. Sfeir et al., 2021).

### Eating behaviors in Lebanon

Within the Lebanese population, a past study indicated a 23.8% prevalence of maladaptive eating behaviors (Haddad et al., 2021). As for orthorexia nervosa in particular, 10.1% of university students in Lebanon exhibited orthorexic tendencies recently (Merhy, Moubarak, Hallit, Obeid, & Hallit, 2023). Unsurprisingly, disordered eating styles are interrelated among Lebanese people: maladaptive eating patterns were significantly associated with orthorexia nervosa (Boustani & Guiné, 2023; Souheil Hallit et al., 2021a). It is imperative to note that eating habits in Lebanon are heavily influenced by environmental factors, most importantly social learning (Boustani & Guiné, 2023).

### Rationale and aim

The current study is guided by a few motivators. First, the research offered in the past implies the significant direct and indirect relationship between cognitive factors and different eating behaviors (Rossi et al., 2024a). Yet, a clear distinction can be made between these eating behaviors despite exhibiting similar interplay with cognitive variables (Rossi, Pietrabissa, Castelnuovo, & Mannarini, 2024). Second, previous investigations show conflicting results regarding eating patterns and sociodemographic factors such as gender, socioeconomic status, and BMI, leaving the literature with inconsistencies. These results prompt further investigation on the topic of dieting associated with strict dietary restrictions specifically and eating patterns overall, in addition to physical and psychological factors that are possibly linked within the Lebanese population. For these reasons, this study evaluates the relationships between different eating patterns, cognitive factors such as rumination and restriction and physical variables including BMI and physical activity, as opposed to normative eating behaviors. This study aimed to identify differentiating elements between maladaptive and normative eating behaviors and cognitions to clarify the existing associations.

## Methods

### Study design

A cross-sectional design was adopted for the current study. It occurred between June and July 2022. Four hundred participants aged 18 and above participated. The research team used a snowball sampling method to recruit volunteers from all governorates of Lebanon. Participants were given access to the survey through a weblink. No remuneration was offered for participation. A pilot study with 20 volunteers (from friends and family members who were not included as participants) was conducted prior to data collection.

### Minimal sample size calculation

Dolnicar et al. (2016) found that the sample size has the strongest effect when it is 10 to 30 times the number of clustering variables, which equals 6 in this study (Three Factor Eating Questionnaire (TFEQ) cognitive restraint, TFEQ uncontrolled eating, TFEQ emotional eating, Repetitive Negative Thinking (RNT) core features, RNT unproductiveness, and RNT mental capacity). Therefore, the minimum sample size should range between 60 and 180.

### Questionnaire

The self-reported questionnaire was in Arabic, one of the primary languages in Lebanon. Its duration was about 20 min. It consisted of multiple parts. Sociodemographic characteristics such as gender, age, marital status, and household crowding index (HCI) (Melki et al., 2004), and the physical activity index (Weary-Smith, 2007) were included in the first section of the measure. Participants provided their weight and height to determine the BMI (World Health Organization). In order to assess financial burden, the following question was asked, “How much pressure do you feel with regard to your personal financial situation in general?”. It was rated on a scale from 1 to 10, with 10 being the highest level of pressure. As for the next section of the survey, it consisted of the subsequent scales:

#### Eating Attitude Test (EAT-26)

This scale is validated in Lebanon (Haddad et al., 2021) and was used to assess maladaptive attitudes related to food (David M. Garner et al., 1982). Good internal validity and reliability were exhibited (David M. Garner et al., 1982). It includes 26 items such as “I avoid eating when I am hungry” and “I feel that food controls my life.” Three subscales exist within the EAT-16: dieting, bulimia and food preoccupation, and oral control. The statements are scored from infrequently/almost never/never (0) to always (3) (F. Fekih-Romdhane, Obeid, Malaeb, Hallit, & Hallit, 2022). Possible disordered eating attitudes are reflected by a score of 20 or above (David M Garner & Garfinkel, 1979). The Cronbach’s alpha values were *α* = 0.91 for the total score, 0.87 for the EAT dieting, 0.83 for the EAT bulimia and food preoccupation, and 0.76 for the EAT oral control subscales.

#### Düsseldorf Orthorexia Scale (DOS)

This scale was validated for Lebanese adults (Souheil Hallit et al., 2021a) and adolescents (Mhanna, Azzi, Hallit, Obeid, & Soufia, 2021) previously. It includes 10 items, which are self-administered, to assess orthorexic eating behaviors. The participants score the items from 1 to 4, with 1 signaling “this does not apply to me” and 4 signaling “this applies to me” (F Barthels, Meyer, & Pietrowsky, 2015). The maximum score is 40; with higher scores indicating more pronounced orthorexic behavior. Some of the items include statements such as “I feel good when I eat healthy food” and “I mainly eat foods that I consider to be healthy” (Friederike Barthels et al., 2019) (*α* = 0.88).

#### Three Factor Eating Questionnaire (TFEQ-R18)

Eighteen items can be found in the TFEQ-R18. This scale, validated in Arabic (Alhebshi et al., 2023), evaluates three domains of eating behaviors: cognitive restraint, uncontrolled eating, and emotional eating (Stunkard & Messick, 1985). The answers are given a value between 1 and 4 (Items 1 to 13, 1 = definitely false and 4 = definitely true; Item 14, 1 = only at meal times and 4 = almost always; Item 15, 1 = almost never and 4 = almost always; Item 16, 1 = unlikely and 4 = very likely; Item 17, 1 = never and 4 = at least once a week; Item 18 is the following *On a scale of 1 to 8, where 1 means no restraint in eating (eating whatever you want, whenever you want it) and 8 means total restraint (constantly limiting food intake and never “giving in”), what number would you give yourself?*) with higher scores indicating greater cognitive restraint, uncontrolled, or emotional eating. Examples of items include “How often do you feel hungry?” and “Do you go on eating binges though you are not hungry?”(Banna et al., 2018). The Cronbach’s alpha values were 0.52 for the TFEQ cognitive restraint, 0.87 for the TFEQ uncontrolled eating, and 0.84 for the TFEQ emotional eating subscales.

#### The Perseverative Thinking Questionnaire (PTQ)

The PTQ (Ehring et al., 2011), validated in Lebanon among adolescents (Feten Fekih-Romdhane et al., 2023), has 15 items that assess elements related to repetitive negative thinking, such as “My thoughts repeat themselves” and “Thoughts intrude into my mind” (Devynck et al., 2017). The answers were scored from 0 indicating “never” to 4 indicating “almost always.” The higher the participants score on each dimension, the higher the level of repetitive negative thinking. The Cronbach’s alpha values were 0.94 for the RNT core features, 0.82 for the RNT unproductiveness, and 0.83 for the RNT mental capacity.

### Ethics approval and consent to participate

The study protocol was approved by the Ethics and Research Committee of the School of Pharmacy at the Lebanese International University. The written consent of each participant was provided upon the submission of the online survey. The methods performed adhered to the appropriate guidelines and regulations.

### Statistical analysis

SPSS version 23 was used to conduct the data analysis (IBM Corp., Armonk, NY, USA). There were no missing values. The scales used were assessed for reliability by calculating Cronbach’s alpha values. The skewness and kurtosis of the dieting and ON scores verified the normal distribution of the sample since they varied between − 1.96 and + 1.96. A hierarchical cluster analysis was conducted based on the Z scores of all scales with the total sample, using Ward’s method with Euclidean distance, due to the study having an exploratory design. It was suggested that Ward’s method would be more suitable for various types of data structures compared to other hierarchical algorithms (Milligan, 1981). As for numerical variables, the Euclidean distance, which is commonly used as a distance measure, was more appropriate (Karna & Gibert, 2022; Yim & Ramdeen, 2015). In order to disclose the naturally occurring subgroups in the sample that are homogenous with regard to highly similar observations they contain but significantly different from each other, the hierarchical cluster analysis was done (Rencher, 2002). Based on information from the agglomeration schedule and dendrogram, the optimal number of clusters was found. More precisely, the agglomeration schedule lists all the successive steps (N-1) that cluster analysis uses to progressively merge clusters with greatest similarity. Adding to that, it shows coefficients to determine the distance of the two clusters being combined at given stages. K-means clustering was done to appoint each individual to the identified clusters after determining the number of clusters (Yakin et al., 2022). One-way ANOVA (Analysis of Variance) was used to compare the cluster groups in terms of dieting, orthorexia nervosa, TFEQ, and RNT subscale scores. We used the independent *t* test to compare the dieting and orthorexia nervosa mean scores between genders and marital status (dichotomous variables). We set the significance level at *p* < 0.05 since it represents a trade-off between type I errors (false positives) and type II errors (false negatives). By setting the risk of error α at 0.05, researchers accept a moderate risk of incorrectly rejecting the null hypothesis while still maintaining a reasonable probability of correctly rejecting it when it is false.

## Results

Four hundred participants filled out the survey (mean age: 25.93 years ± 11.87 [min = 18; max = 84]; 65% females). Table 1 summarizes other sociodemographic characteristics.

**Table 1:** Sociodemographic and other characteristics of the sample (*n* = 400)

Gender	
Male	140 (35.0%)
Female	260 (65.0%)
Marital status	
Single	323 (80.8%)
Married	77 (19.3%)
Age (years)	25.93 (11.87)
Body Mass Index (kg/m^2^)	23.62 (4.48)
Household crowding index (person /room)	1.15 (0.60)
Financial burden	4.78 (2.74)
Physical activity index	24.85 (19.55)
TFEQ cognitive restraint	15.32 (3.66) [median = 16; min = 6; max = 25]
TFEQ uncontrolled eating	18.30 (5.92) [median = 18; min = 9; max = 34]
TFEQ emotional eating	6.49 (2.78) [median = 6; min = 3; max = 12]
RNT core features	15.33 (8.69) [median = 15; min = 0; max = 36]
RNT unproductiveness	4.78 (3.04) [median = 5; min = 0; max = 12]
RNT mental capacity	4.52 (3.15) [median = 4; min = 0; max = 12]

Numbers are shown as n (%) or mean (SD)

### Cluster analysis

The results of the cluster analysis categorized participants into 3 clusters: cluster 1 (maladaptive eating behavior and rumination) *N* = 145), cluster 2 (normative eating behavior and thought; *N* = 145), and cluster 3 (restriction of food intake and rumination) *N* = 110) (Table 2).

**Table 2:** Cluster analysis

	**Cluster 1** Maladaptive eating behavior and rumination **(*N* = 145)**	**Cluster 2** Normative eating behavior and thought **(*N* = 145)**	**Cluster 3** Restriction of food intake and rumination **(*N* = 110)**	**Cluster 1** Maladaptive eating behavior and rumination **(*N* = 145)**	**Cluster 2** Normative eating behavior and thought **(*N* = 145)**	**Cluster 3** Restriction of food intake and rumination **(*N* = 110)**
	**Regression coefficients**			**Mean ± SD**		
TFEQ cognitive restraint	0.38	− 0.50	0.17	16.70 ± 3.22	13.48 ± 3.68	15.93 ± 3.15
TFEQ uncontrolled eating	0.92	− 0.59	-0.43	23.72 ± 4.40	14.82 ± 4.52	15.73 ± 3.73
TFEQ emotional eating	1.00	− 0.65	-0.46	9.27 ± 1.72	4.69 ± 1.98	5.21 ± 1.71
RNT core features	0.60	− 0.97	0.49	20.53 ± 6.62	6.90 ± 4.83	19.57 ± 6.27
RNT unproductiveness	0.63	− 0.98	0.46	6.69 ± 2.40	1.81 ± 1.48	6.18 ± 2.20
RNT mental capacity	0.60	− 0.89	0.38	6.40 ± 2.82	1.72 ± 1.62	5.73 ± 2.41

### Bivariate analysis

A higher mean dieting score was found in participants belonging to cluster 1 compared to the other 2 clusters, whereas a higher mean DOS score was significantly found in men vs women, married vs single, and in participants belonging to cluster 1 compared to the other 2 clusters. Furthermore, higher BMI, TFEQ cognitive restraint, uncontrolled eating, and emotional eating, as well as RNT core features, unproductiveness, and mental capacity, were significantly associated with higher dieting and ON tendencies. Finally, older age was significantly associated with higher ON tendencies (Tables 3 and 4).

**Table 3:** Bivariate analysis of variables associated with dieting and orthorexia scores

Variable	Dieting	*P*	Orthorexia nervosa	*P*
Sex		0.593		**0.007**
Male	7.81 ± 7.70		20.79 ± 7.18	
Female	7.37 ± 7.95		18.86 ± 6.58	
Marital status		0.547		**0.024**
Single/divorced/ widowed	7.41 ± 7.93		19.16 ± 6.82	
Married	8.01 ± 7.56		21.12 ± 6.79	
Clusters		** < 0.001**		** < 0.001**
Cluster 1: maladaptive eating behavior and rumination	10.03 ± 8.17		21.56 ± 6.94	
Cluster 2: normative eating behavior and thought	4.86 ± 6.57		17.39 ± 5.91	
Cluster 3: restriction of food intake and rumination	7.75 ± 7.93		19.70 ± 7.11	

Numbers in bold reflect significant *p* values

**Table 4:** Pearson correlation matrix

	Mean ± SD	1	2	3	4	5	6	7	8	9	10	11	12
1. Orthorexia nervosa	19.54 ± 6.85	1											
2. Dieting	7.53 ± 7.86	0.42***	1										
3. Age	25.93 ± 11.87	0.15**	0.04	1									
4. Body Mass Index	23.62 ± 4.48	0.22***	0.25***	0.34***	1								
5. Household Crowding Index	1.15 ± 0.60	0.01	0.03	− 0.24***	− 0.14**	1							
6. Financial burden	4.78 ± 2.74	0.07	− 0.01	0.15**	0.06	0.09	1						
7. Physical activity index	24.85 ± 19.55	0.13*	0.10	− 0.12*	0.03	0.08	− 0.16**	1					
8. TFEQ cognitive restraint	15.32 ± 3.66	0.54***	0.36***	0.11*	0.23***	− 0.002	0.03	0.07	1				
9. TFEQ uncontrolled eating	18.30 ± 5.92	0.18***	0.15**	− 0.13*	0.18***	0.14**	0.08	− 0.01	0.16**	1			
10. TFEQ emotional eating	6.49 ± 2.78	0.19***	0.17**	− 0.05	0.21***	0.10*	0.06	− 0.01	0.26***	0.68***	1		
11. RNT core features	15.33 ± 8.69	0.18***	0.24***	− 0.18***	0.05	0.07	0.05	− 0.01	0.28***	0.36***	0.41***	1	
12. RNT unproductiveness	4.78 ± 3.04	0.16**	0.23***	− 0.19***	0.04	0.11*	0.08	− 0.01	0.27***	0.38***	0.40***	0.86***	1
13. RNT mental capacity	4.52 ± 3.15	0.18***	0.22***	− 0.14**	0.07	0.14**	0.06	0.02	0.27***	0.35***	0.39***	0.82***	0.83***

*p < 0.05; **p < 0.001; ***p < 0.001

### Multivariable analyses

Higher BMI (Beta = 0.36), and belonging to cluster 3 (Beta = 2.87) and cluster 1 (Beta = 4.50) compared to cluster 2 were significantly associated with more dieting (Table 5, Model 1).

**Table 5:** Multivariable analyses

	**Unstandardized Beta**	**Standardized Beta**	***P***	**95% CI**
**Model 1: Linear regression (using the ENTER method) of factors associated with dieting**				
Body Mass Index	0.36	0.21	** < 0.001**	0.20; 0.53
Physical activity index	0.03	0.08	0.082	− 0.004; 0.07
Cluster 3 vs Cluster 2*	2.87	0.16	**0.002**	1.04; 4.70
Cluster 1 vs Cluster 2*	4.50	0.28	** < 0.001**	2.78; 6.23
**Model 2: Linear regression (using the ENTER method) of factors associated with orthorexia nervosa**				
Age	0.06	0.11	0.144	− 0.02; 0.15
Sex (females vs males*)	− 1.00	− 0.07	0.185	− 2.49; 0.48
Body Mass Index	0.15	0.10	0.063	− 0.01; 0.32
Marital status (married vs single*)	0.61	0.04	0.626	− 1.83; 3.05
Financial burden	0.12	0.05	0.332	− 0.12; 0.36
Physical activity index	0.05	0.13	**0.009**	0.01; 0.08
Cluster 3 vs Cluster 2*	2.48	0.16	**0.003**	0.88; 4.09
Cluster 1 vs Cluster 2*	4.10	0.29	**< 0.001**	2.55; 5.65

*Reference group; numbers in bold indicate significant *p*-values

Higher physical activity index (Beta = 0.05), and belonging to cluster 3 (Beta = 2.48) and cluster 1 (Beta = 4.10) compared to cluster 2 were significantly associated with more ON tendencies (Table 5, Model 2).

## Discussion

The current study’s results show that higher BMI, restriction of food intake and rumination, maladaptive eating behavior, and rumination were significantly related to more dieting compared to normative eating behavior and thought. In addition, higher physical activity index, restriction of food intake and rumination, and maladaptive eating behavior and rumination were significantly related to higher levels of orthorexia nervosa tendencies compared to normative eating behavior and thought.

### BMI, rumination, and eating behaviors

Higher BMI, restriction of food intake and rumination, and maladaptive eating behavior and rumination, compared with normative eating behavior and thought, were significantly associated with more dieting in the current study. First, dietary patterns that do not involve restriction of food intake were associated with lower BMI (Razavi et al., 2021). As for engaging in restrictive diets, women specifically were more likely to increase in BMI in the future (Field et al., 2007). Another study showed that both men and women who dieted in a restrictive or maladaptive way increased in BMI across the span of 10 years (Neumark-Sztainer et al., 2012). Furthermore, among obese individuals, as in those having a high BMI, rumination was associated with maladaptive eating patterns (Wang et al., 2017). Other findings suggest that rumination was connected to both restriction of food intake and maladaptive eating patterns in women specifically (Dondzilo et al., 2016). Dieting and restrictive eating patterns were positively associated with repetitive negative thinking (A Brytek-Matera et al., 2021). Most importantly, rumination related to food restriction was a significant determining factor for maladaptive eating behaviors among women (Soleymani et al., 2022). It is important to note that rumination was proven to be a determining pathway to maladaptive eating patterns (Palmieri et al., 2021). It can be hypothesized that rumination exacerbates restriction of food intake and dieting through repetitive thinking about eating behaviors, which subsequently produces an adverse reaction that worsens maladaptive eating behaviors contributing to more weight gain and higher BMI.

### Physical activity, rumination, and eating behaviors

Higher physical activity index, restriction of food intake and rumination, and maladaptive eating behavior and rumination, compared with normative eating behavior and thought, were significantly associated with more orthorexia nervosa tendencies. Physical activity was recently found to exacerbate rumination, but this investigation was conducted on children (Ling et al., 2023). As for adults, physical activity was only negatively associated with rumination when it was perceived as pleasurable (Kagawa et al., 2022). Our present results are in accordance with recent ones where maladaptive eating patterns and higher intense physical activity were associated with orthorexia nervosa tendencies (Anna Brytek-Matera et al., 2022). Other findings even connected orthorexia nervosa to addiction to physical activity (Rudolph, 2018). Another connection to orthorexia nervosa was rumination, where it served as a predictive factor (Carpita et al., 2022). Furthermore, while previous findings found no orthorexia nervosa cannot predict obsessive–compulsive symptoms (S. Hallit et al., 2022), other authors suggest a possible overlap between the two variables, with OCD involving a prominent component of rumination translated in repetitive intrusive thoughts (Yazkan & Uğurlu, 2022). In a Lebanese sample, it was found that individuals with higher orthorexia nervosa tendencies were more concerned with dieting, which includes restrictive eating patterns, than those with normative eating behaviors (Al Kattan, 2016). In a different sample, orthorexia nervosa tendencies were positively correlated with physical exercise, maladaptive eating patterns, and excessive thought about food intake (Cerolini et al., 2022). Furthermore, orthorexia nervosa behaviors, physical activity, restrictive lifestyle, and dietary choices were interconnected (Brodock et al., 2021). Evidently, the present results are useful as the prevalence of orthorexia nervosa within the Lebanese population is notable: 8.4% in a sample of adults and 10.1% among university students, as previously mentioned (A. Brytek-Matera, Sacre, Staniszewska, & Hallit, 2020). The current knowledge from our study confirms the association between different orthorexia nervosa diagnostic criteria: high physical activity index, restriction of food intake, and maladaptive eating behaviors can be classified into adherence to strict dietary rules and lifestyle behaviors, and rumination can be categorized into excessive preoccupation and focus on maintaining a desired eating style (Cena et al., 2019).

## Limitations and strengths

First, the present study was conducted in a cross-sectional design, meaning the data were collected at only one point in time. Furthermore, we can only infer the presence and strength of relationships between variables and not cause and effect. Second, the cognitive restraint subscale in the TFEQ has a Cronbach’s alpha of 0.52, which only indicates moderate reliability for that specific set of items. Third, the use of self-reported measures in the study increases the possibility of responder bias. Also, the TFEQ and the PTQ are not validated for the Lebanese population. Furthermore, the snowball sampling technique used might increase the probability of selection bias especially that the mean age of our sample is relatively young and females are predominant. The sample size from each governorate was not proportional to the population size, which could hinder the ability to generalize the current results to the general Lebanese population. Having said that, this study has notable strengths. To begin with, all scales used were available in Arabic, the native language of Lebanon. Second, successfully contrasts some of the factors associated with both normative and maladaptive eating patterns. Finally, it opens the door for further research to be conducted on the topic by providing previous findings.

## Clinical implications

Maladaptive eating behaviors, which can be indicative of eating disorders, have detrimental psychological and physical effects. In this study, specific cognitive and physical patterns were connected to certain eating patterns in a sample of Lebanese adults. These results provide the advantage of being able to identify indicators of orthorexia nervosa and constant dieting in order to avoid adverse consequences in the current context. Furthermore, the findings could provide insight on the potential to treat maladaptive eating disorders by adopting cognitive therapies that target rumination. Existing interventions can also be modified to focus on cognitive elements, such as ruminative thoughts, that play an important role in the development and sustainability of maladaptive eating patterns, potentially providing further advancement to Lebanese health services.

## Recommendations

Current results can serve as a valuable baseline for future research. First, it reinforces the importance of conducting longitudinal studies to benefit from findings based on cohort providing data on cognitive factors involved in eating behaviors. Also, it stresses the importance of validating more scales assessing the variables at hand, given the significant associations found.

## Conclusion

The current research shows a relationship between BMI, physical activity, rumination, and eating patterns including restriction of food intake, dieting, and orthorexia nervosa. These results contribute to a growing body of literature about orthorexia nervosa and its associated cognitive and physical factors. It also sheds light on maladaptive thinking patterns that coincide or contribute to dieting and orthorexia nervosa. Additionally, it sheds light on the connection between sociodemographic factors, BMI, and physical activity, with maladaptive eating and cognitive patterns. Further insight is provided on the existing connections related to eating pathologies or potentially harmful eating behaviors within the Lebanese population.

## Data Availability

The dataset is not publicly available but is available upon a reasonable request from the corresponding author (SH).

## Abbreviations

BMI: Body mass index
TFEQ: Three factor eating questionnaire
RNT: Repetitive negative thinking
PTQ: Perseverative thinking questionnaire
DOS: Düsseldorf orthorexia scale
EAT-26: Eating attitude test
HCI: Household crowding index

## References

[CR1] Adan, R. A. H., van der Beek, E. M., Buitelaar, J. K., Cryan, J. F., Hebebrand, J., Higgs, S., . . . Institutionen för neurovetenskap och, f. (2019). Nutritional psychiatry: Towards improving mental health by what you eat. *European neuropsychopharmacology, 29*(12), 1321–1332. 10.1016/j.euroneuro.2019.10.011 10.1016/j.euroneuro.2019.10.011 31735529

[CR2] Al Kattan, M. (2016). The prevalence of orthorexia nervosa in Lebanese university students and the relationship between orthorexia nervosa and body image, body weight and physical activity. University of Chester. Masters dissertation. Available from: https://chesterrep.openrepository.com/handle/10034/620467.

[CR3] Alhebshi, S., Hilary, S., Safi, S. K., Ali, H. I., Ismail, L. C., Al Dhaheri, A., & Stojanovska, L. (2023). Validity and reliability of the Arabic version of the Three-Factor Eating Questionnaire-R18. *Heliyon, 9*(7). e17623. 10.1016/j.heliyon.2023.e17623. PMID: 37455991; PMCID: PMC10345239. 10.1016/j.heliyon.2023.e17623 PMC10345239 37455991

[CR4] Bamia, C., Martimianaki, G., Kritikou, M., & Trichopoulou, A. (2017). Indexes for assessing adherence to a Mediterranean diet from data measured through brief questionnaires: Issues raised from the analysis of a Greek population study. *Current Developments in Nutrition,**1*(3), e000075–e000075. 10.3945/cdn.116.000075 29955694 10.3945/cdn.116.000075 PMC5998912

[CR5] Banna, J., Panizza, C., Boushey, C., Delp, E., & Lim, E. (2018). Association between cognitive restraint, uncontrolled eating, emotional eating and BMI and the amount of food wasted in early adolescent girls. *Nutrients,**10*(9), 1279. 10.3390/nu10091279 30201904 10.3390/nu10091279 PMC6164919

[CR6] Barthels, F., Barrada, J. R., & Roncero, M. (2019). Orthorexia nervosa and healthy orthorexia as new eating styles. *PLoS ONE,**14*(7), e0219609–e0219609. 10.1371/journal.pone.0219609 31291364 10.1371/journal.pone.0219609 PMC6619829

[CR7] Barthels, F., Meyer, F., & Pietrowsky, R. (2015). [The Düsseldorf orthorexia scale – Construction and evaluation of a questionnaire for assessing orthorexic eating behavior]. *J Clin Psychol Psychother, 44*(2). 10.1026/1616-3443/a000310

[CR8] Boucher, J. L. (2017). Mediterranean eating pattern. *Diabetes Spectrum,**30*(2), 72–76. 28588371 10.2337/ds16-0074 PMC5439355

[CR9] Boustani, N. M., & Guiné, R. P. (2023). Food determinants and motivation factors impact on consumer behavior in Lebanon. *Open Agriculture,**8*(1), 20220176.

[CR10] Brodock, J. L., Hayes, J. E., Masterson, T. D., & Hopfer, H. (2021). Differences in preferred fat level, sweetener type, and amount of added sugar in chocolate milk in a choice task relate to physical activity and orthorexia. *Appetite,**163*, 105214. 33771648 10.1016/j.appet.2021.105214

[CR11] Brown, T. A., Forney, K. J., Klein, K. M., Grillot, C., & Keel, P. K. (2020). A 30-year longitudinal study of body weight, dieting, and eating pathology across women and men from late adolescence to later midlife. *Journal of Abnormal Psychology,**129*(4), 376. 32309984 10.1037/abn0000519 PMC7179079

[CR12] Brytek-Matera, A., Bronowicka, P., & Walilko, J. (2021). Restraint theory: Significance of rumination. *European Psychiatry,**64*(S1), S179–S180.

[CR13] Brytek-Matera, A. J. A. o. P., & psychotherapy. (2012). Orthorexia nervosa–an eating disorder, obsessive-compulsive disorder or disturbed eating habit.* 1*(1), 55–60.

[CR14] Brytek-Matera, A., Pardini, S., Szubert, J., & Novara, C. (2022). Orthorexia nervosa and disordered eating attitudes, self-esteem and physical activity among young adults. *Nutrients,**14*(6), 1289. 35334945 10.3390/nu14061289 PMC8948728

[CR15] Brytek-Matera, A., Sacre, H., Staniszewska, A., & Hallit, S. (2020). The prevalence of orthorexia nervosa in Polish and Lebanese adults and its relationship with sociodemographic variables and BMI ranges: A cross-cultural perspective. *Nutrients, 12*(12). 10.3390/nu12123865 10.3390/nu12123865 PMC7767210 33348787

[CR16] Carpita, B., Cremone, I. M., Amatori, G., Cappelli, A., Salerni, A., Massimetti, G., . . . Dell’Osso, L. (2022). Investigating the relationship between orthorexia nervosa and autistic traits in a university population. *CNS spectrums, 27*(5), 613–620. 10.1017/S1092852921000420 33866990

[CR17] Cena, H., Barthels, F., Cuzzolaro, M., Bratman, S., Brytek-Matera, A., Dunn, T., . . . Donini, L. M. (2019). Definition and diagnostic criteria for orthorexia nervosa: A narrative review of the literature. *Eating and weight disorders-studies on anorexia, bulimia and obesity, 24*(2), 209–246. 10.1007/s40519-018-0606-y 30414078

[CR18] Cerolini, S., Vacca, M., Zagaria, A., Donini, L. M., Barbaranelli, C., & Lombardo, C. (2022). Italian adaptation of the Düsseldorf Orthorexia Scale (I-DOS): Psychometric properties and prevalence of orthorexia nervosa among an Italian sample. *Eating and Weight Disorders-Studies on Anorexia, Bulimia and Obesity,**27*(4), 1405–1413. 10.1007/s40519-021-01278-2 PMC9079031 34351591

[CR19] Contois, E. J. H. (2019). Gender and dieting. *Encyclopedia of food and agricultural ethics*, *4*, 1398–1403.

[CR20] Costa, C. B., Hardan-Khalil, K., & Gibbs, K. (2017). Orthorexia nervosa: A review of the literature. *Issues in Mental Health Nursing,**38*(12), 980–988. 10.1080/01612840.2017.1371816 29215937 10.1080/01612840.2017.1371816

[CR21] Devynck, F., Kornacka, M., Baeyens, C., Serra, É., Neves, J. F., & d., Gaudrat, B., … Romo, L. (2017). Perseverative Thinking Questionnaire (PTQ): French validation of a transdiagnostic measure of repetitive negative thinking. *Frontiers in Psychology,**8*, 2159–2159. 10.3389/fpsyg.2017.02159 29326620 10.3389/fpsyg.2017.02159 PMC5733460

[CR22] Dolnicar, S., Grün, B., & Leisch, F. (2016). Increasing sample size compensates for data problems in segmentation studies. *Journal of Business Research,**69*(2), 992–999.

[CR23] Dondzilo, L., Rieger, E., Palermo, R., Byrne, S., & Bell, J. (2016). Association between rumination factors and eating disorder behaviours in young women. *Advances in Eating Disorders,**4*(1), 84–98.

[CR24] Donini LM, Barrada JR, Barthels F, Dunn TM, Babeau C, Brytek-Matera A, Cena H, Cerolini S, Cho HH, Coimbra M, Cuzzolaro M, Ferreira C, Galfano V, Grammatikopoulou MG, Hallit S, Håman L, Hay P, Jimbo M, Lasson C, Lindgren EC, McGregor R, Minnetti M, Mocini E, Obeid S, Oberle CD, Onieva-Zafra MD, Opitz MC, Parra-Fernández ML, Pietrowsky R, Plasonja N, Poggiogalle E, Rigó A, Rodgers RF, Roncero M, Saldaña C, Segura-Garcia C, Setnick J, Shin JY, Spitoni G, Strahler J, Stroebele-Benschop N, Todisco P, Vacca M, Valente M, Varga M, Zagaria A, Zickgraf HF, Reynolds RC, Lombardo C. A consensus document on definition and diagnostic criteria for orthorexia nervosa. Eat Weight Disord. 2022 Dec;27(8):3695-3711. doi: 10.1007/s40519-022-01512-5. Epub 2022 Nov 27. Erratum in: Eat Weight Disord. 2023 Sep 16;28(1):76. 10.1007/s40519-023-01599-4. 10.1007/s40519-022-01512-5 PMC9803763 36436144

[CR25] Ehring, T., Zetsche, U., Weidacker, K., Wahl, K., Schönfeld, S., & Ehlers, A. (2011). The Perseverative Thinking Questionnaire (PTQ): Validation of a content-independent measure of repetitive negative thinking. *Journal of Behavior Therapy and Experimental Psychiatry,**42*(2), 225–232. 21315886 10.1016/j.jbtep.2010.12.003 PMC3042595

[CR26] Fekih-Romdhane, F., Malaeb, D., Sarray El Dine, A., Yakın, E., Hallit, S., & Obeid, S. (2023). Association between bullying victimization and aggression in Lebanese adolescents: The indirect effect of repetitive negative thinking—A path analysis approach and scales validation. *Children,**10*(3), 598. 36980156 10.3390/children10030598 PMC10047793

[CR27] Fekih-Romdhane, F., Obeid, S., Malaeb, D., Hallit, R., & Hallit, S. (2022). Validation of a shortened version of the Eating Attitude Test (EAT-7) in the Arabic language. *Journal of Eating Disorders,**10*(1), 127. 10.1186/s40337-022-00651-5 36028891 10.1186/s40337-022-00651-5 PMC9412802

[CR28] Field, A. E., Aneja, P., Austin, S. B., Shrier, L. A., de Moor, C., & Gordon-Larsen, P. (2007). Race and Gender differences in the association of dieting and gains in BMI among young adults. *Obesity (Silver Spring, Md.), 15*(2), 456–464. 10.1038/oby.2007.560 10.1038/oby.2007.560 17299119

[CR29] Garner, D. M., & Garfinkel, P. E. (1979). The Eating Attitudes Test: An index of the symptoms of anorexia nervosa. *Psychological Medicine,**9*(2), 273–279. 472072 10.1017/s0033291700030762

[CR30] Garner, D. M., Olmsted, M. P., Bohr, Y., & Garfinkel, P. E. (1982). The Eating Attitudes Test: Psychometric features and clinical correlates. *Psychological Medicine,**12*(4), 871–878. 10.1017/S0033291700049163 6961471 10.1017/s0033291700049163

[CR31] Gedeon, R., Hallit, S., & Wakim, L. H. (2022). Food insecurity and eating habits of Lebanese children aged 5–11 years during the COVID-19 pandemic and the socioeconomic crisis: A national study. *BMC Public Health,**22*(1), 1982. 10.1186/s12889-022-14387-z 36307806 10.1186/s12889-022-14387-z PMC9616611

[CR32] Grzymisławska, M., Puch, E. A., Zawada, A., & Grzymisławski, M. (2020). Do nutritional behaviors depend on biological sex and cultural gender? *Advances in Clinical & Experimental Medicine, 29*(1), 165–172. 10.17219/acem/111817. PMID: 32017478. 10.17219/acem/111817 32017478

[CR33] Haddad, C., Khoury, C., Salameh, P., Sacre, H., Hallit, R., Kheir, N., . . . Hallit, S. (2021). Validation of the Arabic version of the Eating Attitude Test in Lebanon: A population study. *Public Health Nutrition, 24*(13), 4132–4143. 10.1017/S1368980020002955 PMC10195249 32895080

[CR34] Hallit, S., Azzi, V., Malaeb, D., & Obeid, S. (2022). Any overlap between orthorexia nervosa and obsessive-compulsive disorder in Lebanese adults? Results of a cross-sectional study and validation of the 12-item and 4-item obsessive-compulsive inventory (OCI-12 and OCI-4). *BMC Psychiatry,**22*(1), 470. 10.1186/s12888-022-04119-3 35836242 10.1186/s12888-022-04119-3 PMC9282139

[CR35] Hallit, S., Barrada, J. R., Salameh, P., Sacre, H., Roncero, M., & Obeid, S. (2021a). The relation of orthorexia with lifestyle habits: Arabic versions of the Eating Habits Questionnaire and the Dusseldorf Orthorexia Scale. *Journal of Eating Disorders,**9*(1), 1–12. 34391484 10.1186/s40337-021-00455-z PMC8364025

[CR36] Kagawa, F., Yokoyama, S., Takamura, M., Takagaki, K., Mitsuyama, Y., Shimizu, A., . . . Okamoto, Y. (2022). Decreased physical activity with subjective pleasure is associated with avoidance behaviors. *Scientific reports, 12*(1), 2832–2832. 10.1038/s41598-022-06563-3 10.1038/s41598-022-06563-3 PMC8857298 35181696

[CR37] Karna, A., & Gibert, K. (2022). Automatic identification of the number of clusters in hierarchical clustering. *Neural Computing and Applications,**34*(1), 119–134.

[CR38] Lee, H.-J., Park, S., Kim, C.-i., Choi, D.-w., Lee, J. S., Oh, S. M., . . . Oh, S. W. (2013). The association between disturbed eating behavior and socioeconomic status: The Online Korean Adolescent Panel Survey (OnKAPS). *PLoS One, 8*(3), e57880. 10.1371/journal.pone.0057880 PMC3589486 23472117

[CR39] Levinson, J. A., Sarda, V., Sonneville, K., Calzo, J. P., Ambwani, S., & Austin, S. B. (2020). Diet pill and laxative use for weight control and subsequent incident eating disorder in US young women: 2001–2016. *American Journal of Public Health,**110*(1), 109–111. 31751147 10.2105/AJPH.2019.305390 PMC6893330

[CR40] Ling, F. C. M., Simmons, J., & Horton, M. (2023). Development and Validation of Physical Activity-Specific Rumination Scale for Children through UK children’s voice. *Research Quarterly for Exercise and Sport,**94*(1), 283–293. 10.1080/02701367.2021.1971150 35344472 10.1080/02701367.2021.1971150

[CR41] Markey, C. H., & Meghan M. Gillen. (2023). Dieting. In *Encyclopedia of Mental Health (pp. 662–668). Academic Press.*

[CR42] Mei, D., Deng, Y., Li, Q., Lin, Z., Jiang, H., Zhang, J., . . . Yan, G. (2022). Current status and influencing factors of eating behavior in residents at the age of 18~ 60: A cross-sectional study in China. *Nutrients, 14*(13), 2585. 10.3390/nu14132585 PMC9268282 35807764

[CR43] Melki, I., Beydoun, H., Khogali, M., Tamim, H., & Yunis, K. (2004). Household crowding index: A correlate of socioeconomic status and inter-pregnancy spacing in an urban setting. *Journal of Epidemiology & Community Health,**58*(6), 476–480. 15143115 10.1136/jech.2003.012690 PMC1732777

[CR44] Merhy, G., Moubarak, V., Hallit, R., Obeid, S., & Hallit, S. (2023). The indirect role of orthorexia nervosa and eating attitudes in the association between perfectionism and muscle dysmorphic disorder in Lebanese male University students–Results of a pilot study. *BMC Psychiatry,**23*(1), 55. 36670380 10.1186/s12888-023-04549-7 PMC9854036

[CR45] Mhanna M, Azzi R, Hallit S, Obeid S, Soufia M. (2022 )Validation of the Arabic version of the Teruel Orthorexia Scale (TOS) among Lebanese adolescents. *Eating and Weight Disorders-Studies on Anorexia, Bulimia and Obesity*. *27*(2), 619-627. 10.1007/s40519-021-01200-w. Epub 2021 May 3. PMID: 33939127; PMCID: PMC8091151. 10.1007/s40519-021-01200-w PMC8091151 33939127

[CR46] Milligan, G. W. (1981). A review of Monte Carlo tests of cluster analysis. *Multivariate Behavioral Research,**16*(3), 379–407. 26815599 10.1207/s15327906mbr1603_7

[CR47] Monteagudo, C., Mariscal-Arcas, M., Rivas, A., Lorenzo-Tovar, M. L., Tur, J. A., & Olea-Serrano, F. (2015). Proposal of a Mediterranean diet serving score. *PLoS ONE,**10*(6), e0128594–e0128594. 10.1371/journal.pone.0128594 26035442 10.1371/journal.pone.0128594 PMC4452755

[CR48] Morrissey, R. A., Gondoli, D. M., & Corning, A. F. (2020). Reexamining the restraint pathway as a conditional process among adolescent girls: When does dieting link body dissatisfaction to bulimia? *Development and Psychopathology,**32*(3), 1031–1043. 31658908 10.1017/S0954579419001287

[CR49] Neumark-Sztainer, D. P. D. M. P. H. R. D., Wall, M. P. D., Story, M. P. D., & Standish, A. R. B. S. (2012). Dieting and unhealthy weight control behaviors during adolescence: Associations with 10-year changes in body mass index. *Journal of Adolescent Health,**50*(1), 80–86. 10.1016/j.jadohealth.2011.05.010 10.1016/j.jadohealth.2011.05.010 PMC3245517 22188838

[CR50] Niedzielski, A., & Kaźmierczak-Wojtaś, N. (2021). Prevalence of orthorexia nervosa and its diagnostic tools—A literature review. *International Journal of Environmental Research and Public Health,**18*(10), 5488. 34065506 10.3390/ijerph18105488 PMC8160773

[CR51] Nolen-Hoeksema, S., & Watkins, E. R. (2011). A heuristic for developing transdiagnostic models of psychopathology: Explaining multifinality and divergent trajectories. *Perspectives on Psychological Science,**6*(6), 589–609. 10.1177/1745691611419672 26168379 10.1177/1745691611419672

[CR52] Palmieri, S., Mansueto, G., Scaini, S., Caselli, G., Sapuppo, W., Spada, M. M., . . . Ruggiero, G. M. (2021). Repetitive negative thinking and eating disorders: A meta-analysis of the role of worry and rumination. *Journal of clinical medicine, 10*(11), 2448. 10.3390/jcm10112448 PMC8198834 34073087

[CR53] Plichta, M., & Jezewska-Zychowicz, M. (2020). Orthorexic tendency and eating disorders symptoms in Polish students: Examining differences in eating behaviors. *Nutrients,**12*(1), 218. 31952161 10.3390/nu12010218 PMC7019551

[CR54] Radwan, H., Hasan, H. A., Ismat, H., Hakim, H., Khalid, H., Al-Fityani, L., . . . Ayman, A. (2019). Body mass index perception, body image dissatisfaction and their relations with weight-related behaviors among university students. *International Journal of Environmental Research and Public Health, 16*(9), 1541. 10.3390/ijerph16091541 PMC6539402 31052368

[CR55] Razavi, R., Parvaresh, A., Abbasi, B., Yaghoobloo, K., Hassanzadeh, A., Mohammadifard, N., . . . Safavi, S. M. (2021). The alternate-day fasting diet is a more effective approach than a calorie restriction diet on weight loss and hs-CRP levels. *Int J Vitam Nutr Res, 91*(3–4). 10.1024/0300-9831/a000623 10.1024/0300-9831/a000623 32003649

[CR56] Rencher, A. C. (2005) "*A review of “methods of multivariate analysis*. 1083–1085.

[CR57] Rossi, A. A., Mannarini, S., Donini, L. M., Castelnuovo, G., Simpson, S., & Pietrabissa, G. (2024a). Dieting, obsessive-compulsive thoughts, and orthorexia nervosa: Assessing the mediating role of worries about food through a structural equation model approach. *Appetite,**193*, 107164. 38103790 10.1016/j.appet.2023.107164

[CR58] Rossi, A. A., Pietrabissa, G., Castelnuovo, G., & Mannarini, S. (2024). Cognitive restraint, uncontrolled eating, and emotional eating. The Italian version of the Three Factor Eating Questionnaire-Revised 18 (TFEQ-R-18): A three-step validation study. *Eating and weight disorders-studies on anorexia, bulimia and obesity, 29*(1), 16. 10.1007/s40519-024-01642-y PMC10894126 38402372

[CR59] Rudolph, S. (2018). The connection between exercise addiction and orthorexia nervosa in German fitness sports. *Eating and Weight Disorders-Studies on Anorexia, Bulimia and Obesity,**23*, 581–586. 10.1007/s40519-017-0437-2 28884261

[CR60] Sfeir E, Haddad C, Salameh P, Sacre H, Hallit R, Akel M, Honein K, Akiki M, Kheir N, Obeid S, Hallit S. Binge eating, orthorexia nervosa, restrained eating, and quality of life: a population study in Lebanon. Eat Weight Disord. 2021 Feb;26(1):145-158. doi: 10.1007/s40519-019-00831-4. 10.1007/s40519-019-00831-4 31849002

[CR61] Sfeir M, Malaeb D, Obeid S, Hallit S. Association between religiosity and orthorexia nervosa with the mediating role of self-esteem among a sample of the Lebanese population - short communication. J Eat Disord. 2022 Oct 24;10(1):151. doi: 10.1186/s40337-022-00672-0. 36280860 10.1186/s40337-022-00672-0 PMC9589842

[CR62] Smith, K. E., Mason, T. B., & Lavender, J. M. (2018). Rumination and eating disorder psychopathology: A meta-analysis. *Clinical Psychology Review,**61*, 9–23. 29703429 10.1016/j.cpr.2018.03.004 PMC6462404

[CR63] Soleymani, A., Mazidi, M., Neimeijer, R., & de Jong, P. J. (2022). Eating disorder-specific rumination moderates the association between attentional bias to high-calorie foods and eating disorder symptoms: Evidence from a reliable free-viewing eye-tracking task. *Appetite,**171*, 105934. 35051543 10.1016/j.appet.2022.105934

[CR64] Stunkard, A. J., & Messick, S. (1985). The three-factor eating questionnaire to measure dietary restraint, disinhibition and hunger. *Journal of Psychosomatic Research,**29*(1), 71–83. 3981480 10.1016/0022-3999(85)90010-8

[CR65] Varga, M., Dukay-Szabo, S., & Tury, F. (2013). Orthorexia nervosa and its background factors. *Ideggyogyaszati Szemle,**66*(7–8), 220–227. 23971352

[CR66] Waliłko, J., Bronowicka, P., He, J., & Brytek-Matera, A. (2021). Dieting and disinhibited eating patterns in adult women with normal body weight: Does rumination matter? *Nutrients,**13*(7), 2475. 34371984 10.3390/nu13072475 PMC8308631

[CR67] Wang, S. B., Lydecker, J. A., & Grilo, C. M. (2017). Rumination in patients with binge-eating disorder and obesity: Associations with eating-disorder psychopathology and weight-bias internalization: Rumination in patients with BED and obesity. *European Eating Disorders Review,**25*(2), 98–103. 10.1002/erv.2499. 28078784 10.1002/erv.2499 PMC5318238

[CR68] Watkins, E. R. (2008). Constructive and unconstructive repetitive thought. *Psychological Bulletin,**134*(2), 163. 18298268 10.1037/0033-2909.134.2.163 PMC2672052

[CR69] Watkins, E. R., & Roberts, H. (2020). Reflecting on rumination: Consequences, causes, mechanisms and treatment of rumination. *Behaviour Research and Therapy,**127*, 103573. 32087393 10.1016/j.brat.2020.103573

[CR70] Weary-Smith, K. A. (2007). *Validation of the physical activity index (PAI) as a measure of total activity load and total kilocalorie expenditure during submaximal treadmill walking.* (Doctoral dissertation, University of Pittsburgh).

[CR71] World Health Organization. Body mass index- BMI. Available from: https://www.euro.who.int/en/health-topics/disease-prevention/nutrition/a-healthy-lifestyle/body-mass-index-bmi.

[CR72] Yakın E, Obeid S, Fekih-Romdhane F, Soufia M, Sawma T, Samaha S, Mhanna M, Azzi R, Mina A, Hallit S. "In-between orthorexia" profile: the co-occurrence of pathological and healthy orthorexia among male and female non-clinical adolescents. J Eat Disord. 2022 Nov 3;10(1):155. doi: 10.1186/s40337-022-00673-z. 10.1186/s40337-022-00673-z PMC9633027 36329509

[CR73] Yang, Y. X., Wang, X. L., Leong, P. M., Zhang, H. M., Yang, X. G., Kong, L. Z., . . . Su, Y. X. (2018). New Chinese dietary guidelines: Healthy eating patterns and food-based dietary recommendations. *Asia Pacific Journal of Clinical Nutrition, 27*(4), 908–913. 10.6133/apjcn.072018.03 30045438

[CR74] Yazkan, G., & Uğurlu, N. (2022). The relationship between orthorexia nervosa tendencies and OCD symptoms in healthcare professionals. *J Psych Nursing, 13*(1). 10.14744/phd.2021.87369

[CR75] Yilmaz, Z., Gottfredson, N. C., Zerwas, S. C., Bulik, C. M., & Micali, N. (2019). Developmental premorbid body mass index trajectories of adolescents with eating disorders in a longitudinal population cohort. *Journal of the American Academy of Child & Adolescent Psychiatry,**58*(2), 191–199. 30738546 10.1016/j.jaac.2018.11.008 PMC6766404

[CR76] Yim, O., & Ramdeen, K. T. (2015). Hierarchical cluster analysis: Comparison of three linkage measures and application to psychological data. *The Quantitative Methods for Psychology,**11*(1), 8–21.

